# Supplementary material for: Bioinformatic and fine-scale chromosomal mapping reveal the nature and evolution of eliminated chromosomes in the Japanese hagfish, Eptatretus burgeri, through analysis of repetitive DNA families
Source: PLoS One. 2023 Aug 28;18(8):e0286941. doi: 10.1371/journal.pone.0286941 (PMC10461843; doi:10.1371/journal.pone.0286941)
Supplement: S1 Fig — (PDF) [file pone.0286941.s001.pdf]

Fig 1a

Family 0

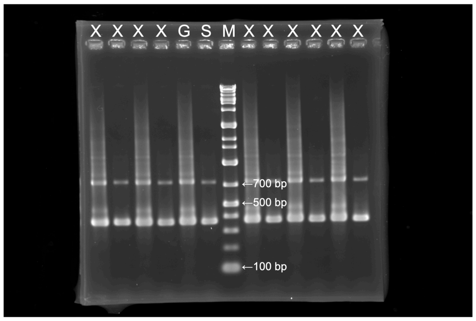

Family 38

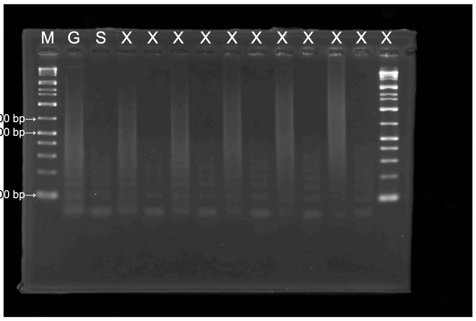

Family 10

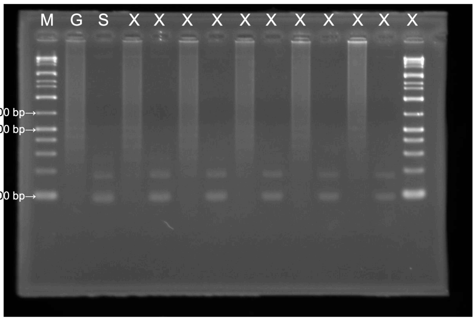

Family 5

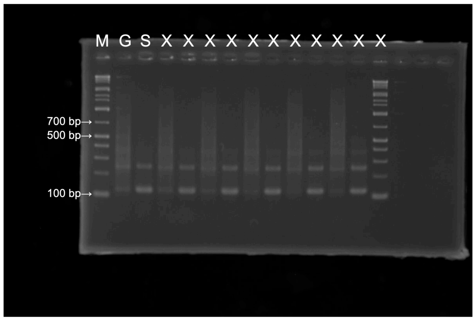

Figure S1. Uncropped and unprocessed original versions of gels shown in Fig 1a. Lanes not included in the figure are marked with X.
